# Assessment of biological dosimetric margin for stereotactic body radiation therapy

**DOI:** 10.1002/acm2.12843

**Published:** 2020-03-06

**Authors:** Daisuke Kawahara, Akito Saito, Shuichi Ozawa, Takehiro Shiinoki, Tomoki Kimura, Kento Tsubouchi, Yasushi Nagata

**Affiliations:** ^1^ Department of Radiation Oncology Institute of Biomedical & Health Sciences Hiroshima University Hiroshima Japan; ^2^ Hiroshima High‐Precision Radiotherapy Cancer Center Hiroshima Japan; ^3^ Department of Radiation Oncology Graduate School of Medicine Yamaguchi University Yamaguchi Japan; ^4^ Section of Radiation Therapy Department of Clinical Support Hiroshima University Hospital Hiroshima Japan

**Keywords:** biological equivalent dose, dosimetric margin, LQ model, SBRT

## Abstract

**Purpose:**

To develop a novel biological dosimetric margin (BDM) and to create a biological conversion factor (BCF) that compensates for the difference between physical dosimetric margin (PDM) and BDM, which provides a novel scheme of a direct estimation of the BDM from the physical dose (PD) distribution.

**Methods:**

The offset to isocenter was applied in 1‐mm steps along left‐right (LR), anterior‐posterior (AP), and cranio‐caudal (CC) directions for 10 treatment plans of lung stereotactic body radiation therapy (SBRT) with a prescribed dose of 48 Gy. These plans were recalculated to biological equivalent dose (BED) by the linear‐quadratic model for the dose per fraction (DPF) of *d* = 3–20 Gy/fr and α/β=3-10. BDM and PDM were defined so that the region that satisfied that the dose covering 95% (or 98%) of the clinical target volume was greater than or equal to the 90% of the prescribed PD and BED, respectively. An empirical formula of the BCF was created as a function of the DPF.

**Results:**

There was no significant difference between LR and AP directions for neither the PDM nor BDM. On the other hand, BDM and PDM in the CC direction were significantly larger than in the other directions. BCFs of *D*_95%_ and *D*_98%_ were derived for the transverse (LR and AP) and longitudinal (CC) directions.

**Conclusions:**

A novel scheme to directly estimate the BDM using the BCF was developed. This technique is expected to enable the BED‐based SBRT treatment planning using PD‐based treatment planning systems.

## 1 Introduction

In modern radiation therapy, dose‐volume histogram (DVH) and isodose distribution are commonly used for treatment evaluation. Dose‐volume constraints indicate organ volumes that should not receive doses exceeding certain limits derived from retrospective studies. Clinical and radiobiological studies have shown that two treatments delivering the same total dose through different fractionation schemes produce different biological results.^1^, ^2^ Fowler showed that the biological effective dose (BED) modeling is a valuable tool for understanding tumor and normal tissue response across different treatment modalities and fractionation schemes.^3^ Based on the idea of BED, it has been shown that the relative biological effectiveness depends on the dose per fraction (DPF) and the number of fractions. Particularly, stereotactic body radiation therapy (SBRT) requires the calculation of BED, as it uses hypo‐fractionations and results in the delivery of a high BED. In these studies, linear quadratic (LQ) model was used. The LQ model is the most commonly used tool to model the effect of fractionation in conventionally fractionated radiotherapy and to predict tumor response to altered fractionation regimens.

Technical advances in radiation therapy, including three‐dimensional conformal radiotherapy (3D‐CRT) and, more recently, intensity‐modulated radiotherapy (IMRT) has simultaneously enabled dose escalation and enhanced normal tissue sparing. ICRU 50 and 62 Reports were widely used as an international reference for the prescribing, recording, and reporting of photon beam radiotherapy.^4^, ^5^ However, the ICRU 83 Report was released in 2010 specifically addressing IMRT and introducing some different concepts and plan evaluation parameters such as volumetric planning target volume (PTV) prescribing.^6^ For SBRT, clinical trials RTOG 0236^7^ and 0813^8^ used the volume prescription method.^9^ The dose that covered 95 % of the PTV (D95%) was conformal in the treatment plan using the volume prescription.^10^, ^11^ The variation of the peripheral dose of PTV is expected to be significantly reduced using this prescribing method.

To accommodate inter‐ and intra‐fractional patient setup uncertainties and organ motions, the International Commission on Radiation Units and Measurements, Inc. (ICRU) recommends expanding the clinical target volume (CTV) by a margin to obtain PTV.^12^ In past studies, the van Herk formula was generally used for calculating the PTV margin from the systematic and random errors of the CTV. This formula ensures that the minimum dose of the CTV is equal to or greater than 95% of the prescribed dose for 90% of the population.^13^ However, the treated volume (TV) is usually larger than the PTV, resulting in a mismatch between the theory and application of the van Herk formula.

Gordon and Siebers introduced a new concept, termed the dosimetric margin (DM), to explain the sensitivity of a group of prostate IMRT treatment plans to patient setup errors.^14^ The TV was defined as a volume covered by the minimum dose of the PTV. The DM, which is a margin achieved between the CTV and TV for a given plan, is a generalization of the conformity index.^15^ Importantly, the sensitivity of the CTV dose to setup errors is a function of the DM. Thus, the target coverage by the isodose surface of interest (e.g., *D*_95%_) should be evaluated using the DM, rather than the CTV‐to‐PTV margin, in the presence of setup errors. However, the DM does not consider the difference in dose distribution by setup uncertainty. Moreover, the DM proposed by Gordon was defined using only the physical dose (PD) calculation. In practice, there are many fractionation schemes for SBRT (e.g., 48 Gy/4 fr, 60 Gy/3 fr etc.). Therefore, it is considered to be essential to take the biological effect such as DPF into account to provide appropriate DM for each fractionation scheme.

In this study, we introduced a DM involving the effects of the dose perturbation due to the setup uncertainty to take into account the setup errors in the clinical practice. The DM was defined as the isocenter shift that the CTV is satisfied with a certain dose level by setup uncertainty. The DM with physical dose distribution is defined as the physical dosimetric margin (PDM). Moreover, we proposed a novel quantity, named biological dosimetric margin (BDM), which was a margin distribution considering the biological effect of the DM. The biological effect was introduced by calculating the BED using the LQ model^16^ as an example biological model. The differences between the relative dose distribution of the PD and BED were calculated. The relative BED distribution was analyzed for the dose per fraction (DPF) from 3 to 20 Gy/fr. The α/β of the tumor and normal tissue were used different values. The α/β of the PTV includes the tumor was 3, 5, and 10 Gy, and that of the normal tissue was 3 Gy.^17^, ^18^, ^19^, ^20^ To provide appropriate DM for each fractionation scheme in BED‐based treatment planning, a biological conversion factor (BCF) between BDM and PDM was introduced by considering the DPF and α/β to create a simple model of the BDM.

## 2 Materials and Methods

Ten cases of patients with lung cancer, who underwent SBRT at (institution name), were analyzed. The characteristics of the patients and their tumors are presented in Table 1. The use of clinical materials in this study was approved by the Institutional Review Board of (institution name).

**Table 1:** Patient characteristics.

Age (years)	Median	78
	Range	58–90
Gender	Male	7 (70%)
	Female	3 (30%)
Tumor location	Right lobe	6 (60%)
	Left lobe	4 (40%)
Tumor diameter (mm)	0–10	3 (30%)
	10–20	4 (40%)
	20–30	3 (30%)

**Fig. 1:**
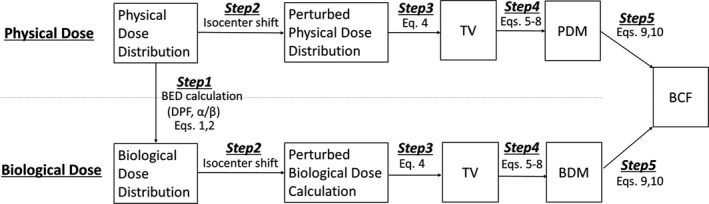
The process of the evaluating the biological dosimetric margin (BDM) and physical dosimetric margin (PDM).

Figure 1 shows the process of the evaluating the BDM and PDM. The physical dose distribution was created in RayStation (RaySearch, Stockholm, Sweden). The physical dose distribution was converted to the BED distribution using the LQ model (Step 1). The dose distribution with the setup uncertainty was created using the “perturbed dose calculation” in RayStation, which the isocenter is shifted from −20 to 20 mm along left‐right (LR), anterior‐posterior (AP), cranio‐caudal (CC) directions (Step 2). These calculations were performed for both physical and biological dose distributions. TV were then derived from the perturbed dose distributions. The anisotropic PDM and BDM were calculated from CTV and TV (Step 3). The BCF model was developed to provide a conversion from the PDM to the BDM (Step 4).

The details of the treatment planning and BED are described in Sections 2.1 and 2.2, respectively. The perturbed dose calculation, TV, and DM are described in detail in Section 2.3. The evaluations of the BDM, PDM, and BCF are given in Section 2.4. In addition, the dose gradient in the physical dose and BED was investigated in Section 2.5.

### 2.1 Treatment planning

All patients were immobilized using a Vac‐Lok cushion (CIVCO, Kalona, IA, USA). Breath‐holding was coordinated in the expiratory phase using Abches (APEX Medical, Tokyo, Japan) — a device that allowed patients to control their chest and abdominal respiratory motion.^21^ The tumor position reproducibility during several expiratory breath‐hold intervals was verified to be within 5 mm using X‐ray fluoroscopy. Computed tomography (CT) scans were performed during the expiratory breath‐holding using a CT scanner (LightSpeed RT16, GE Healthcare, Little Chalfont, UK). Both the slice thickness and the slice interval were 1.25 mm. The diameter of the tumor was equal to or smaller than 3 cm in the clinical cases investigated in this study.

The CTV margin was 0 mm around the GTV. The reproducibility of the tumor position at respiratory breath‐hold was suppressed within 5 mm using the breath‐hold technique with Abches.^22^ Systematic error of the tumor position is corrected with the daily Cone‐beam CT. A PTV margin of 5 mm in left‐right (LR), anterior‐posterior (AP), and cranio‐caudal (CC) directions around the CTV including the respiratory motion reproducibility and the setup error was usually added. The isocenter (IC) was defined at the centroid of the GTV. Eight beams with coplanar and noncoplanar angles were used for every patient. If possible, the beam directions were set such that the beams did not cross the critical OARs, such as the contralateral lung and spinal cord. The dose constraint for normal lung was the combined percentage of lung volume receiving a dose of 20 Gy or higher (*V*_20 Gy_) is below 20%. The dose constraint for the spinal cord was the maximum dose is below 25 Gy. A TrueBeam linear accelerator (Varian Medical Systems, Palo Alto, USA) was used for producing 6‐MV flattening filter free beams. The treatment plans with a prescribed dose of 48 Gy for *D*_95%_ of the PTV was created using the superposition/convolution algorithm on RayStation.

### 2.2 Biological equivalent dose

The BED was calculated using the LQ model as an example model to create the BED. The LQ model fits the cell‐surviving fraction through a second‐order polynomial on the DPF,

(1) $$\text{Cell}\;-\;\text{surviving fraction}\;=\;\exp\left(-\alpha d-\beta d^{2}\right),$$

where *d* is the DPF. The BED is then defined by

(2) $$\text{BED}=nd\left(1+\frac{d}{\alpha /\beta }\right),$$

where *n* is the number of treatment fractions. The ratio α/β describes the repair capacity of the cells, and thus the sensitivity to the fractionation. In the calculation of relative BED, DPF, and α/β were mainly affected with a constant DPF. The BED distribution was calculated from the physical dose distribution using Eq. (2). In this study, α/β was varied along 3, 5, and 10.^23^ The DPF was in the range 3–20 Gy, referring to the past clinical trials shown in Table 2.

**Table 2:** The past clinical trials for lung SBRT that used different DPF in the range 3–20 Gy.

References	Total dose (Gy)	Daily dose (Gy)	Prescription
Shien et al.^24^	33–50	3–5	50–60% margin
Onimaru et al.^25^	48–60	6–7.5	Point dose
Uematsu et al.^26^	50–60	10	80% margin
Nagata et al.^27^	48	12	Point dose
Taremi et al.^28^	48	12	80% margin
Wulf et al.^29^	45–56.2	15–15.4	80% margin
Olsen et al.^30^	50, 54	10, 18	80% margin
Timmerman et al.^31^	24–60	8–20	80% margin

### 2.3 Treated volume and dosimetric margin

**Fig. 2:**
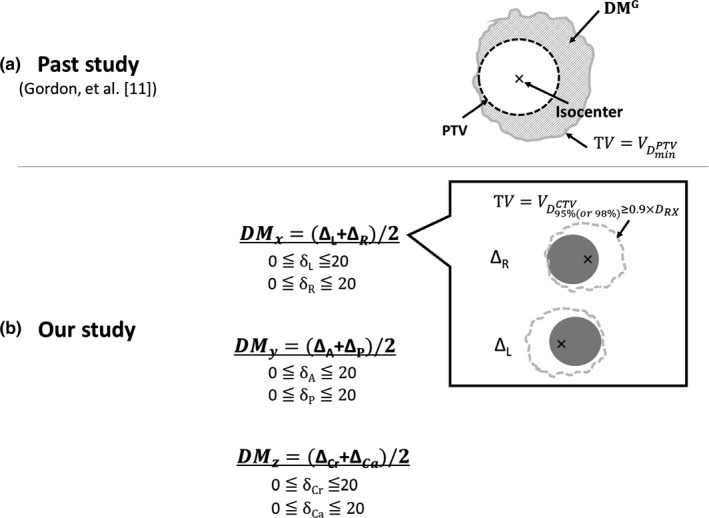
Illustrations of the treated volume (TV) and dosimetric margin (DM) by (a) Gordon and Siebers^14^ and (b) our study. See text for details.

Figure 2 shows illustrations of the TV and DM in this study in comparison with the ones by Gordon and Siebers.^14^ The TV by Gordon and Siebers [Fig. 2(a)] is defined by

(3) $${\text{TV}}^{G}=V_{D_{\min}^{\text{PTV}}},$$

where $D_{X}^{\text{ROI}}$ denotes the dose to the region of interest (ROI) and X = min., max., 95%, etc. The DM by Gordon and Siebers^14^ (${\text{DM}}^{G}$) was then defined as a volume achieved between the CTV and TV. These definitions are given based on a treatment plan with no blurring of the isocenter.

In this study, the TV was defined so that the dose perturbation effects due to the setup error were taken into account [Fig. 2(b)]. The setup error was generated by shifting the isocenter (IC) along LR, AP, and CC directions from −20 to +20 mm with a 1‐mm step ($\delta_{L}$, $\delta_{R}$, $\delta_{A}$, $\delta_{P}$, $\delta_{\text{Cr}}$, and $\delta_{\text{Ca}}$, respectively). The dose distributions were calculated with the shifted isocenter. The TV is defined as a volume that satisfied

(4) $$\text{TV}=V_{D_{95\%\;\left(\text{or}\;98\%\right)}^{\text{CTV}}\;\geq \;0.9\;\times \;D_{\text{Rx}}},$$

where $D_{\text{Rx}}$ denotes the prescribed dose. The $D_{X}^{\text{ROI}}$ denotes the dose to the region of interest (ROI) and X = 95%, 98% in the physical and biological dose distributions by shifting the isocenter. Next, the maximum shift toward the left, right, anterior, posterior, cranio, and caudal directions ($\Delta_{L}$, $\Delta_{R}$, $\Delta_{A}$, $\Delta_{P}$, $\Delta_{\text{Cr}}$, and $\Delta_{\text{Ca}}$, respectively) that passed criteria of Eq. (5) were determined.

(5) $$D_{95\%\;\left(\text{or}\;98\%\right)}^{\text{CTV}}\;\geq \;0.9\;\times \;D_{\text{Rx}}.$$

Here, the scale of the DM in this study that is the distance and the DM^G^ that is the volume are different. The DM along each direction was calculated by

(6) $${\text{DM}}_{x}=\left(\Delta_{L}+\Delta_{R}\right)/2,$$

(7) $${\text{DM}}_{y}=\left(\Delta_{A}+\Delta_{P}\right)/2,\;\text{and}$$

(8) $${\text{DM}}_{z}=\left(\Delta_{\text{Cr}}+\Delta_{\text{Ca}}\right)/2,.$$

Thus, the DM in this study, $\left({\text{DM}}_{x},\;{\text{DM}}_{y},\;{\text{DM}}_{z}\right)$, is anisotropic which includes the dose perturbation effects induced by the isocenter shift. The DM was calculated for both the physical and biological dose distributions.

### 2.4 Evaluations of BDM and PDM

The DM in physical dose distribution is defined as PDM and the DM in BED is defined as BDM. The physical dose distribution and BED are defined as the BDM and PDM. The mean value and standard error of the mean (SEM) of the BDM and PDM of the 10 cases were evaluated for DPF from 3 to 20 Gy/fr. The data were compared using Student's *t*‐test. The first test was performed to compare the BDM and PDM along the LR, AP and CC directions. The LQ model was applied with α/β fixed to 10 Gy, as it is universally accepted for conventional fractionation.^32^ The second test was performed to compare the BDM and PDM with α/β = 3, 5, and 10 Gy. All statistical analyses were performed using SPSS Statistics (IBM, Armonk, NY, USA). The statistical significance was defined for the *P* < 0.05. Also, the correlation factor (r^2^) of the tumor volume and PDM or BDM is analyzed. Then, The BCF was defined as the ratio between the BDM and PDM:

(9) $$\text{BCF}=\frac{\text{BDM}}{\text{PDM}}.$$

The correlation of the BCF and α/β is evaluated. After confirming there is no significant difference for the BCF due to the α/β, the BCF is fitted using the following function of $d/\left(\alpha /\beta \right)$.

(10) $$\text{BCF}=A\;\exp\left[-{\left(\frac{d}{\alpha /\beta }\right)}^{B}\right]+C,$$

where *A*, *B*, and C are the fitting parameters determined by a least squares method. These parameters were determined for the measurement data of *D*_95%_ and *D*_98%_ in the LR, AP, and CC directions.

### 2.5 Evaluations of dose gradient in physical dose and BED

**Fig. 3:**
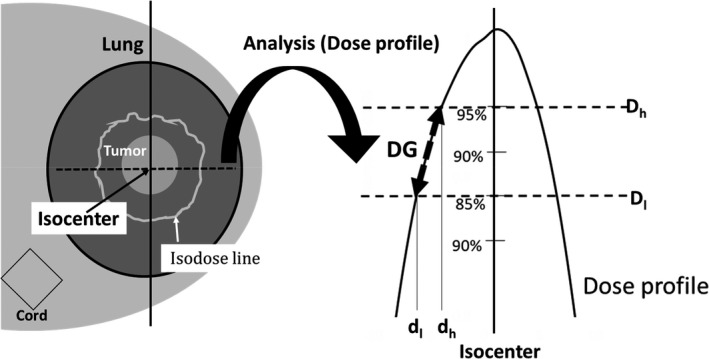
The scheme of the lung tumor and isodose line (left) and the dose gradient (DG) in the dose profile (right). The *d_l_* and *d_h_* are the distance from the isocenter at *D_l_* and *D_h_* that are higher and lower dose. The *D_l_* and *D_h_* are the 85% and 95% of the prescribed dose.

The dose gradient (DG) was calculated with dose profile in the physical dose and BED, as shown in Fig. 3. The dose distribution was normalized to the prescribed dose as 100%. The distance between the *d_h_* and *d_l_* that were the position at a certain relative dose of higher dose (*D_h_*) (%) and lower dose (*D_l_*) (%). The DG can be defined by

(11) $$\text{DG}=\left|\frac{D_{h}-D_{l}}{d_{h}-d_{l}}\right|,$$

The average DG in the LR, AP, and CC directions were defined as ${\text{DG}}_{\text{LR}}$, ${\text{DG}}_{\text{AP}}$, and ${\text{DG}}_{\text{CC}}$, respectively.

(12) $${\text{DG}}_{\text{LR}}=\left({\text{DG}}_{R}+{\text{DG}}_{L}\right)/2,$$

(13) $${\text{DG}}_{\text{AP}}=\left({\text{DG}}_{A}+{\text{DG}}_{P}\right)/2,\;\text{and}$$

(14) $${\text{DG}}_{\text{CC}}=\left({\text{DG}}_{\text{Cr}}+{\text{DG}}_{\text{Ca}}\right)/2.$$

Then, the DG in physical dose distribution (${\text{DG}}^{\text{PD}}$) and the DG in BED (${\text{DG}}^{\text{BED}}$) were derived by

(15) $${\text{DG}}^{\text{PD}}=\left({\text{DG}}_{\text{LR}}^{\text{PD}}\;+\;{\text{DG}}_{\text{AP}}^{\text{PD}}\;+\;{\text{DG}}_{\text{CC}}^{\text{PD}}\right)/3,$$

(16) $${\text{DG}}^{\text{BED}}=\left({\text{DG}}_{\text{LR}}^{\text{BED}}\;+\;{\text{DG}}_{\text{AP}}^{\text{BED}}\;+\;{\text{DG}}_{\text{CC}}^{\text{BED}}\right)/3,$$

## 3 Results

### 3.1 Dose gradient in physical dose and BED

**Fig. 4:**
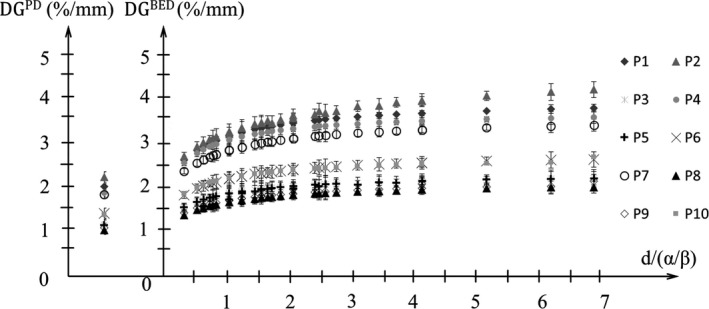
The DG^PD^ and DG^BED^ with the $d/\left(\alpha /\beta \right)$. The dose per fraction (DPF) ranged from 3 to 20 Gy with α/β = 10 Gy.

Figure 4 shows the average DG^PD^ and DG^BED^ for the all directions in 10 patients. The average DG^PD^ of 10 patients was 1.7%/mm and DG^BED^ at the DPF of 3–20 Gy with α/β = 10 Gy of 10 patients were 2.4%–2.9%/mm. The DG^PD^ is significantly smaller than the DG^BED^ at the DPF of 3–20 Gy with α/β = 10 Gy.

### 3.2 Comparison of the physical and biological dosimetric margins

**Fig. 5:**
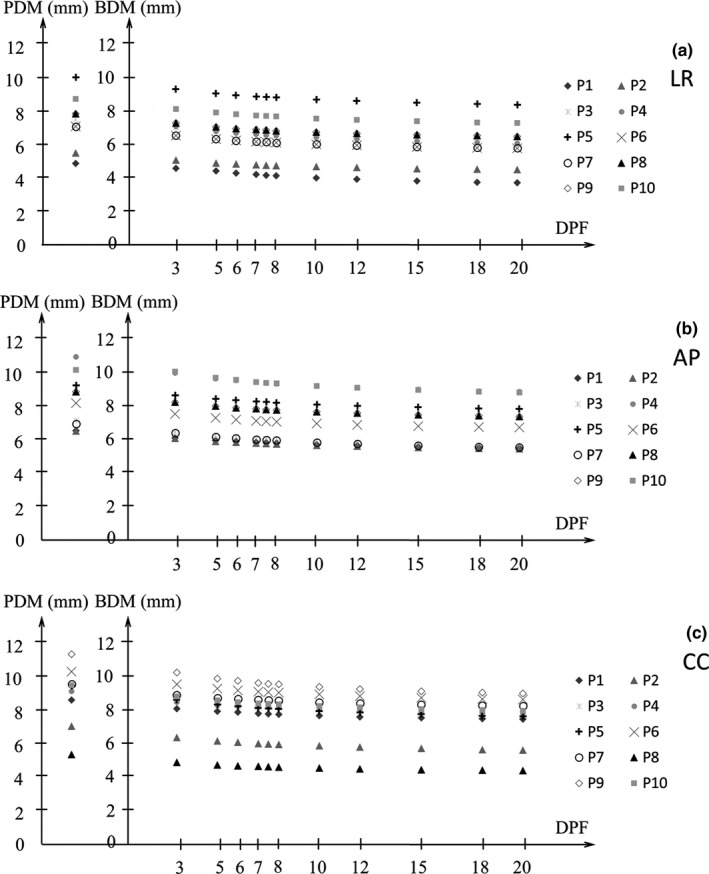
Measured biological dosimetric margin (BDM) and physical dosimetric margin (PDM; the margin corresponding to the 90% coverage of the planned *D*_95%_ of clinical target volume [CTV] with biological equivalent dose [BED] and physical dose [PD] distribution) at the dose per fraction (DPF) of 3–20 Gy with α/β = 10 Gy in (a) LR, (b) AP, and (c) CC directions. AP, anterior‐posterior; CC, cranio‐caudal; LR, left‐right.

**Fig. 6:**
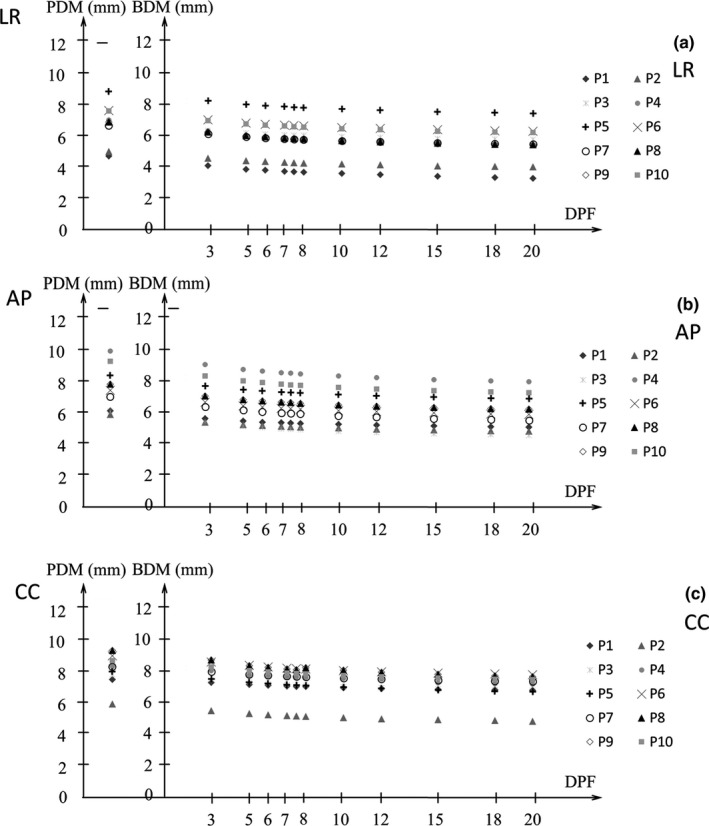
Measured biological dosimetric margin (BDM) and physical dosimetric margin (PDM) (the margin corresponding to the 90% coverage of the planned *D*_98%_ of clinical target volume [CTV] with biological equivalent dose [BED] and physical dose [PD] distribution) at the dose per fraction (DPF) of 3–20 Gy with α/β = 10 Gy in (a) LR, (b) AP, and (c) CC directions. AP, anterior‐posterior; CC, cranio‐caudal; LR, left‐right.

Figures 5 and 6 show the BDM and PDM (α/β = 10 Gy) for the $D_{95\%}^{\text{CTV}}$ and $D_{98\%}^{\text{CTV}}$ of CTV in the LR, AP, and CC directions for all patients. The DPF ranged from 3 to 20 Gy. The difference between the BDM and PDM of *D*_95%_ was 0.5–1.3, 0.6–1.4, and 0.6–1.3 mm in the LR, AP, and CC directions, respectively (Fig. 5). The difference between the BDM and PDM of *D*_98%_ was 0.5–1.2, 0.6–1.3, and 0.5–1.2 mm for the LR, AP, and CC directions, respectively (Fig. 6). The PDM was larger than the BDM, and the BDM was smaller for large DPF in all directions. There was no significant difference in LR and AP directions for neither the BDM nor PDM (*P *> 0.05). On the other hand, both the BDM and PDM in the CC direction were significantly larger than the ones in the other directions (*P* < 0.05) in both *D*_95%_ and *D*_98%_.

### 3.3 Correlation of the tumor volume and the DM

**Fig. 7:**
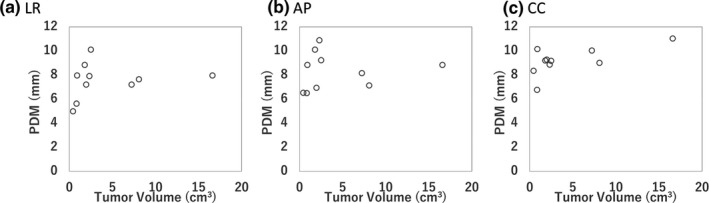
The correlation of the tumor volume and the physical dosimetric margin (PDM) of the $D_{95\%}^{\text{CTV}}$ in (a) LR, (b) AP, (c) CC directions. AP, anterior‐posterior; CC, cranio‐caudal; LR, left‐right.

**Fig. 8:**
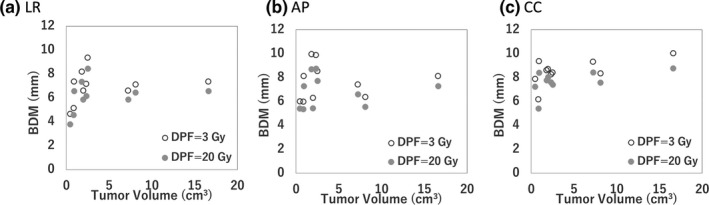
The correlation of the tumor volume and the biological dosimetric margin (BDM) of the $D_{95\%}^{\text{CTV}}$ with 10 Gy of α/β in (a) LR, (b) AP, (c) CC directions. AP, anterior‐posterior; CC, cranio‐caudal; LR, left‐right.

Figure 7 shows the correlation of the tumor volume and PDM for the $D_{95\%}^{\text{CTV}}$ of CTV in the LR, AP, and CC directions. The r^2^ for the tumor volume and PDM in LR, AP, and CC directions were 0.18, 0.07, and 0.64, respectively. Figure 8 shows the correlation of the tumor volume and BDM (α/β = 10 Gy) for the $D_{95\%}^{\text{CTV}}$ of CTV with *DPR* = 3 and 20 Gy in the LR, AP, and CC directions. The r^2^ for the tumor volume and BDM with *DPR* = 3 Gy in LR, AP, and CC directions were 0.18, 0.03, and 0.59, respectively. The r^2^ for the tumor volume and BDM with *DPR* = 20 Gy in LR, AP, and CC directions were 0.20, 0.04, and 0.42, respectively.

### 3.4 Biological conversion factor

**Fig. 9:**
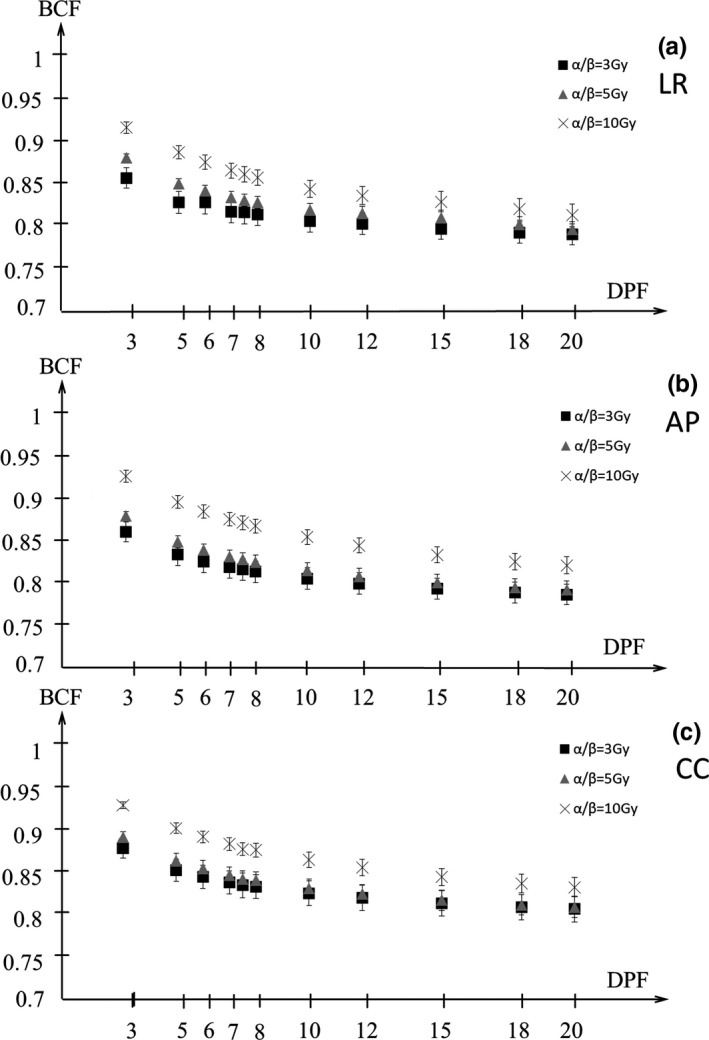
The biological conversion factor (BCF) of *D*_95%_ at the dose per fraction (DPF) of 3–20 Gy with α/β = 3, 5, 10 Gy in the (a) LR, (b) AP, and (c) CC directions. Closed squares, closed triangles, and cross symbols show the BCFs for α/β = 3, 5, 10 Gy, respectively. AP, anterior‐posterior; CC, cranio‐caudal; LR, left‐right.

**Fig. 10:**
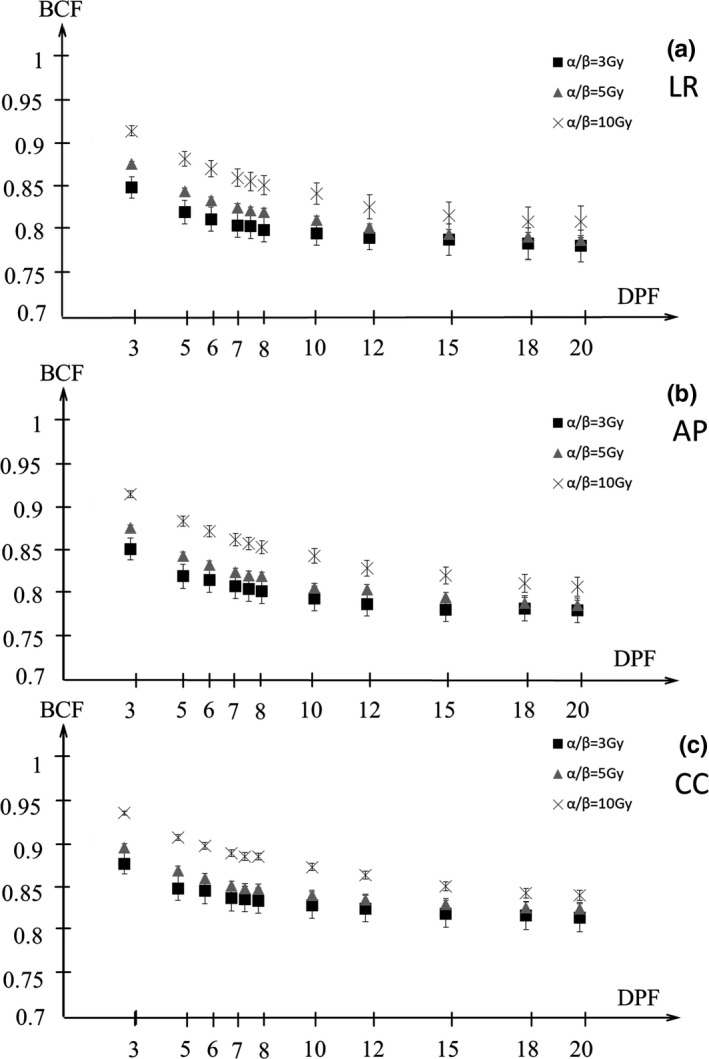
The biological conversion factor (BCF) of *D*_98%_ at the dose per fraction (DPF) of 3–20 Gy with α/β = 3, 5, 10 Gy in the (a) LR, (b) AP, and (c) CC directions. Closed squares, closed triangles, and cross symbols show the BCFs for α/β = 3, 5, 10 Gy, respectively. AP, anterior‐posterior; CC, cranio‐caudal; LR, left‐right.

**Fig. 11:**
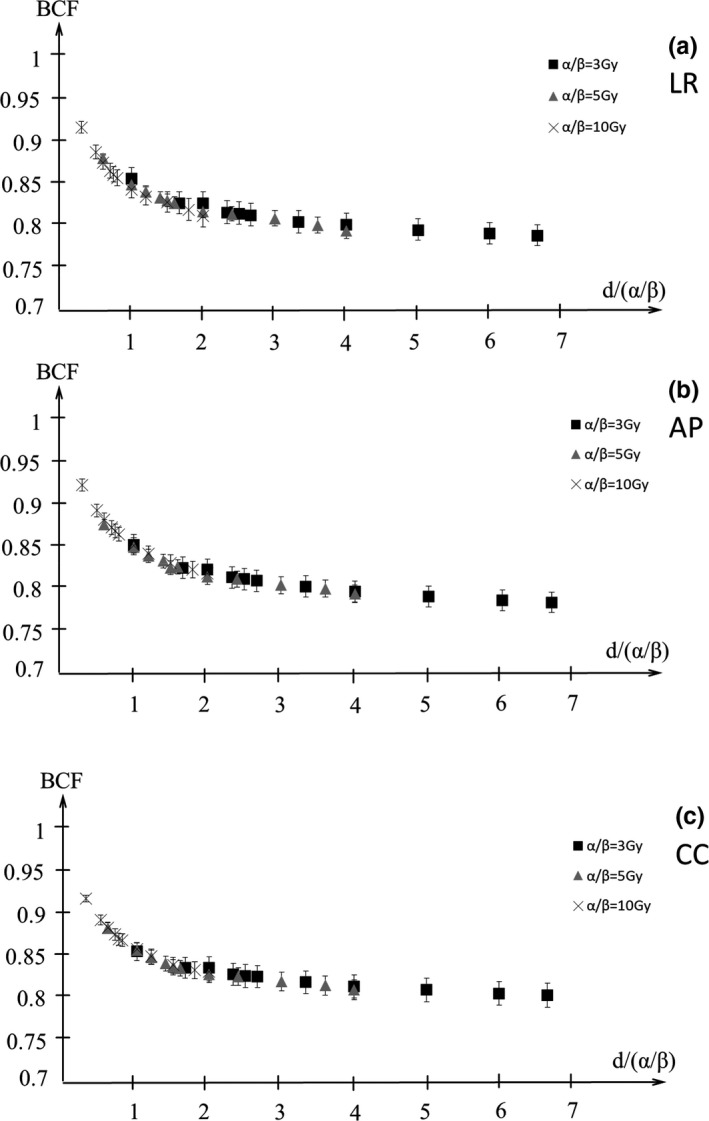
The biological conversion factor (BCF) of *D*_98%_ with the $d/\left(\alpha /\beta \right)$ in the (a) LR, (b) AP, and (c) CC directions. Closed squares, closed triangles, and cross symbols show the BCFs for α/β = 3, 5, 10 Gy, respectively. AP, anterior‐posterior; CC, cranio‐caudal; LR, left‐right.

**Fig. 12:**
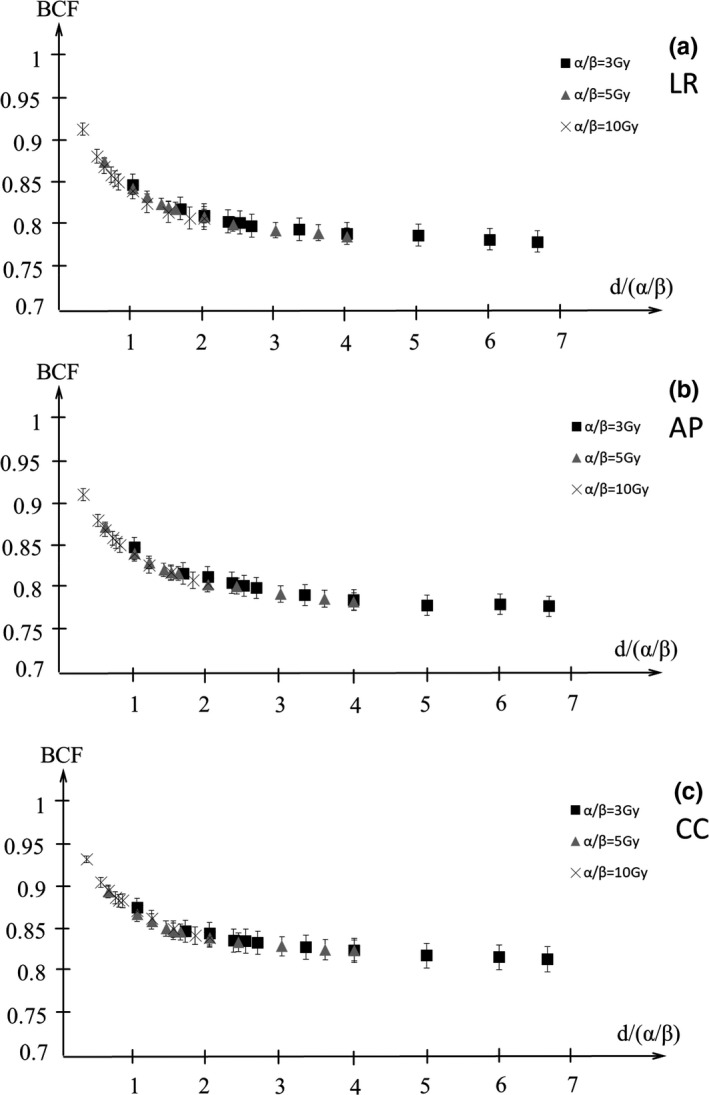
The biological conversion factor (BCF) of *D*_98%_ with the $d/\left(\alpha /\beta \right)$ in the (a) LR, (b) AP, and (c) CC directions. Closed squares, closed triangles, and cross symbols show the BCFs for α/β = 3, 5, 10 Gy, respectively. AP, anterior‐posterior; CC, cranio‐caudal; LR, left‐right.

**Fig. 13:**
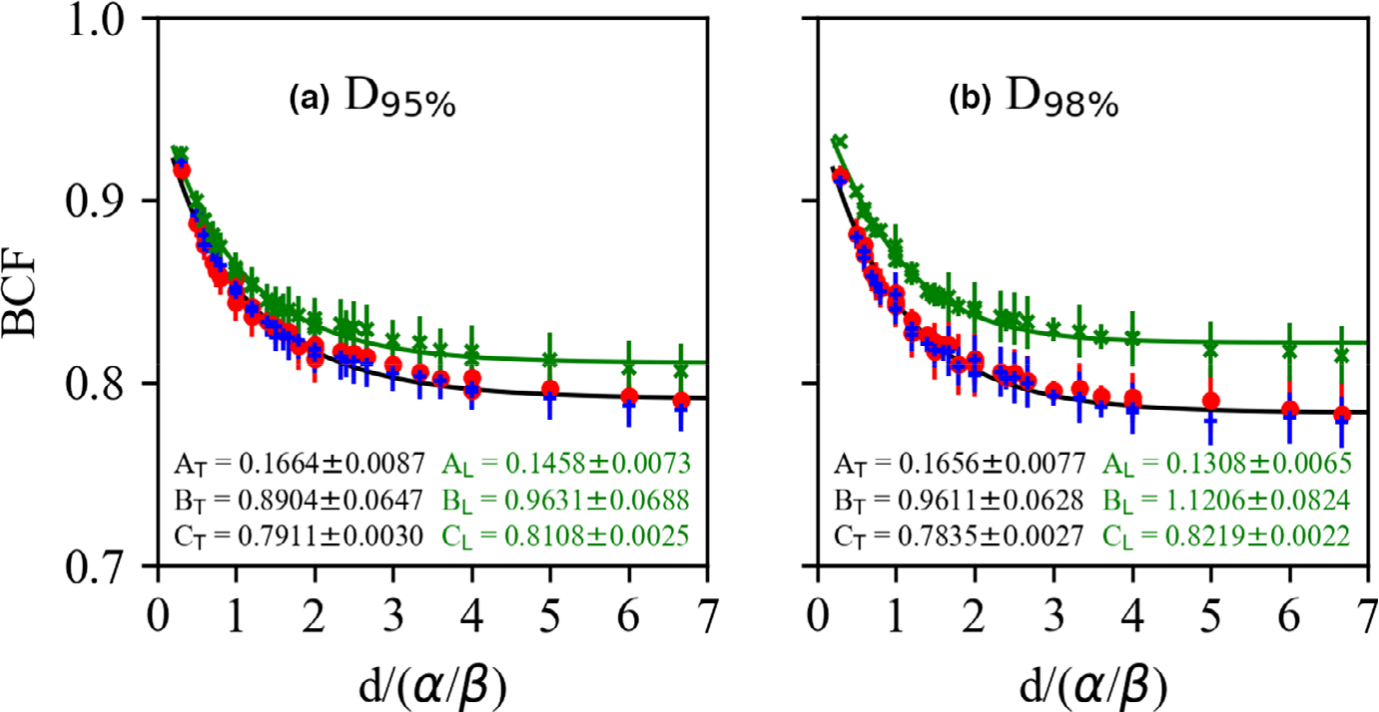
The biological conversion factor (BCF) of the (a) *D*_95%_ and (b) *D*_98%_ with the $d/\left(\alpha /\beta \right)$. Closed red circles, plus symbols, and cross symbols are the data of the BCF along the LR, AP, and CC directions. Error bars represent the standard error of the mean. Solid black and green curves are the results of the fitting using Eq. (10) for the transverse (LR and AP) and longitudinal (CC) data. The *A_T_*, *B_T_*, and *C_T_* are fitting parameters for the transverse direction, and the *A_L_*, *B_L_,* and *C_L_* are fitting parameters for the longitudinal direction. AP, anterior‐posterior; CC, cranio‐caudal; LR, left‐right.

Figures 9 and 10 show the BCF for the $D_{95\%}^{\text{CTV}}$ and $D_{98\%}^{\text{CTV}}$ of CTV with α/β = 3, 5, 10 Gy in the LR, AP, and CC directions. The BCF is smaller with higher DPF and lower α/β. Figures 11 and 12 show the BCF for the $D_{95\%}^{\text{CTV}}$ and $D_{98\%}^{\text{CTV}}$ of CTV with $d/\left(\alpha /\beta \right)$ in the LR, AP, and CC directions. The differences in the BCF due to α/β for the $D_{95\%}^{\text{CTV}}$ and $D_{98\%}^{\text{CTV}}$ of CTV were not significantly. The data of the transverse direction (LR and AP directions) were combined for the fitting since there was no significant difference between the LR and AP directions. The fitting results of the BCF are shown in Fig. 13. Figure 13(a) shows the measurement data and the fitted curve of *D*_95%_ in the transverse and CC directions, respectively. Figure 13(b) show the measurement data and the fitted curve of *D*_98%_ in the transverse and CC directions, respectively. The resulting parameters of the BCF are shown in Fig. 13.

## 4 Discussion

In a past study, van Herk reported that the PTV should be a geometrical concept, and van Herk's margin was defined to select the appropriate beam sizes and arrangements, taking into consideration the net effect of all the possible geometrical variations and inaccuracies to obtain a clinically acceptable and specified probability that the prescribed dose is absorbed in the CTV.^13^ Gordon and Siebers reported the use of the DM, which extended the concept of the CTV‐to‐PTV margin.^14^ The DM by Gordon was defined as the distance between the CTV and the region of the minimum PTV dose. These concepts were introduced as an isotropic margin from the CTV. On the other hand, the DM along the LR, AP and CC directions were independently defined in this study. The DM in the CC direction was found to be larger than the other directions. This result is an indication that the anisotropic nature of the DM introduced in this study would be useful to provide an appropriate 3D margin. The importance of the anisotropic margin is supported by the results in Caivano et al.^33^ They reported that the CT with a thin slice thickness could be suggested for small targets, such as those treatable with the stereotactic radiotherapy, to achieve a better tumor definition and dose coverage. The resolution of the dose coverage of the target depends on the slice thickness in the CC direction. Thus, it would be beneficial to use the anisotropic DM to create a reasonable 3D dose distribution with a minimum 3D margin. Moreover, the correlation between the tumor volume and DM (BDM and PDM) was evaluated. This correlation is weaker, which indicates that the size of the target volume does not affect the BDM and PDM in this study.

There are various patient positioning uncertainties such as respiratory motion, the breath‐hold reproducibility, contouring, and residual set up errors for SBRT treatment.^34^, ^35^ The manifestation of the isocenter shift depends on the type of the immobilization, respiratory gating or breath‐holding, and irradiation techniques. While the dose perturbation due to the setup error was mimicked by systematically moving the isocenter along the LR, AP, and CC directions in this study, the BCF can be adjusted to give a dedicated model for specific technique by replacing the 3D isocenter shift by the actual distribution of the setup error.

The essential outcome of this study is the novel scheme to involve the biological effects into the DM. The BED suggested in past studies^3^, ^24^, ^25^, ^26^, ^27^, ^28^, ^29^, ^30^, ^31^ has been an important subject to consider the difference in the prescribed dose. Specifically, the SBRT is performed with various fractionations and prescribed doses at different institutions. The difference in the DPF affects the biological damage. While the treatment plan review is usually performed on the PD in the clinical practice, it is essential to consider the difference in the DPF. The BDM was smaller than PDM in all directions for the DPFs examined through the comparison between the BDM and PDM in this study. The BDM is smaller with larger DPF and higher α/β. It was affected by the dose gradient. The dose gradient in physical dose was significantly smaller than that in the BED at the $d/\left(\alpha /\beta \right)$ examined through the comparison. Also, the dose gradient in the BED is lager with higher $d/\left(\alpha /\beta \right)$. The plan in the BED with higher $d/\left(\alpha /\beta \right)$ is less forgiving of set‐up inaccuracies owing to steep dose gradients. Therefore, in order to give the appropriate BDM in the BED‐based treatment planning, the dependency of the BDM on the DPF and α/β should be taken into account.

**Fig. 14:**
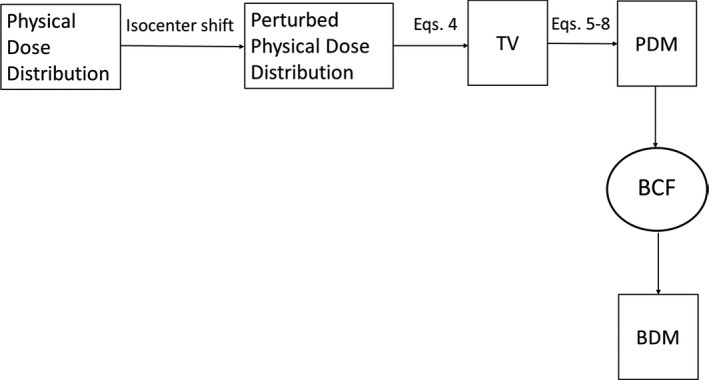
The flowchart of the clinical introduction of the biological conversion factor (BCF).

This study introduced a new index, the BCF, to convert the PDM to BDM. Since the BDM and PDM in the CC direction were larger than the transverse direction, the BCF model was developed separately for the transverse and longitudinal directions. For the clinical introduction of the BCF, the flowchart is shown as Fig. 14. Using the BCF, a direct estimation of the BDM is possible without calculating the BED. Namely, the BCF provides the opportunities to give the biologically equivalent DM used in the current PD‐based treatment planning in the SBRT clinical practice without built‐in functions of the biological conversion of the dose distribution. In the clinical process, at first, the PDM is calculated obtained in commercially treatment planning. After that, the BDM can be obtained without built‐in functions of the biological conversion of the dose distribution. Additionally, the optimal BDM for the BED when the dose per fraction is changed can also be derived.

The simple LQ model is not considered the proliferation and repair of tumors during the whole course of treatment. However, it should be noted that the concept of the BCF is not specific for the LQ model. There were several proposed models for SBRT therapeutic schemes except for LQ model, such as the Linear‐Quadratic‐Linear (LQL) model, the modified Linear‐Quadratic (MQL) model, the generalized Linear‐Quadratic (gLQ) model.^36^, ^37^, ^38^ The model of the BCF can be applied to these biological models and various tumor types through the procedure developed in this study.

The BCF developed in this study can be introduced in the SBRT clinical practice with the current PD‐based treatment planning system with no built‐in BED‐related functions. In practice, the PDM is determined using the SBRT treatment plans for the commissioning. The BDM is then obtained using the BCF using Eqs. (9 and 10). This study analyzed 10 treatment plans and the BCF had variation. Further studies should be needed to reduce the variation. However, this study provides a new framework to give the BDM in the current PD‐based treatment planning system. This novel scheme can be used as a substitute method of the BED‐based treatment planning in the current PD‐based treatment planning system.

The limitation of this study was that the BDM of only one combination of the treatment technique and the treatment site was evaluated with limited number of patients. The accuracy of the BDM for lung cancer and the other cancers will be evaluated in the further study.

## 5 Conclusion

A novel scheme for the direct estimation of the BDM from the PD distribution was developed in this study. The setup error was taken into account for the DM used in this study. The effects of the DPF and α/β were involved into the BCF which provided the direct conversion from the PDF to BDM. This scheme is applicable for the various prescribed doses and fractionations. It is also possible to replace the BCF by replacing the LQ model by some other biological model. The BCF model is useful for evaluating the BED coverage to the target volume, which plays an equivalent role of the BED‐based treatment planning of SBRT in the current PD‐based treatment planning system.

## Conflict of Interest

None.
